# Supplementary material for: Distinctive Deposition Patterns of Sporadic Transthyretin-Derived Amyloidosis in the Atria: A Forensic Autopsy-Based Study
Source: Int J Mol Sci. 2024 Jul 26;25(15):8176. doi: 10.3390/ijms25158176 (PMC11311325; doi:10.3390/ijms25158176)
Supplement: Supplementary file 1 [file ijms-25-08176-s001.zip › ijms-3116151-supplementary.pdf]

Online Supplementary Data

Distinctive deposition patterns of sporadic transthyretin-derived amyloidosis in the atria: A forensic autopsy-based study

Shojiro Ichimata[1], Yukiko Hata[1], Koji Yoshida[1], Keiichi Hirono[2], Naoki Nishida[1]

[1] Department of Legal Medicine, Faculty of Medicine, University of Toyama, Japan

[2] Department of Pediatrics, Faculty of Medicine, University of Toyama, Japan

List of Supplementary material

Supplementary Table: 3

Supplementary Table S1. Summary of semi-quantitative histopathological investigation results

	Transthyretin																										Atrial natriuretic factor					
	VS			AS				RA			RAA			LA			LAA			SVC			SAN	AVN	Neu*	GC	AS	RAA	RA	LAA	LA	SVC
#	E	V	I	ER	EL	V	I	E	V	I	E	V	I	E	V	I	E	V	I	E	V	I										
1	0	0	1	0	0	0	1	1	0	1	1	0	1	1	1	1	1	0	1	0	0	1	Neg	Neg	Neg	Neg	0	3	2	3	2	0
2	0	0	3	4	1	1	4	3	2	1	2	0	1	2	1	3	1	1	4	2	0	3	Neg	Neg	Neg	Neg	2	2	2	3	2	1
3	0	0	1	0	0	0	0	0	0	1	0	0	0	0	0	1	0	0	1	0	0	1	Neg	Neg	Neg	Neg	2	2	1	3	2	0
4	0	0	2	0	0	0	0	0	0	1	0	0	1	0	0	1	0	0	1	0	0	0	Neg	Neg	Neg	Neg	2	1	2	2	2	0
5	0	1	3	2	3	0	3	1	0	2	2	1	2	2	1	3	1	0	2	2	0	1	Pos	Neg	Neg	Neg	3	2	2	4	3	0
6	0	2	3	4	1	2	2	1	2	1	1	4	1	2	4	3	1	2	2	3	1	3	Neg	Neg	Pos	Neg	2	3	2	2	2	1
7	0	0	3	2	0	0	2	2	1	1	1	0	1	1	1	2	1	1	2	3	0	1	Neg	Neg	Neg	Neg	3	3	3	3	4	2
8	1	1	3	3	0	1	3	0	0	0	0	0	0	3	1	4	4	1	3	0	0	1	Neg	Neg	Equ	Pos	1	2	1	2	1	1
9	0	0	1	1	0	0	1	0	0	0	0	0	0	0	0	1	0	0	1	1	0	1	Neg	Neg	Neg	Neg	1	2	1	3	2	1
10	1	3	4	4	3	1	4	3	3	4	3	4	2	3	3	4	3	3	3	4	2	3	Pos	Neg	Equ	Neg	4	2	2	3	2	1
11	0	1	2	1	0	0	1	0	0	0	0	0	0	2	1	4	0	0	1	1	0	1	Neg	Neg	Equ	Neg	0	0	0	1	0	0
12	1	2	4	4	1	1	4	1	1	1	1	1	1	2	1	4	2	1	2	4	0	2	Neg	Neg	Neg	Neg	1	2	2	2	2	0
13	0	3	0	4	0	1	4	0	1	0	0	2	0	2	2	2	1	3	4	4	2	4	Neg	Neg	Neg	Pos	0	2	1	3	3	1
14	0	4	1	0	0	3	0	0	3	0	0	3	0	2	3	1	0	4	1	0	3	0	Neg	Neg	Neg	Neg	2	1	1	2	3	1
15	0	0	2	4	0	0	2	1	2	1	4	1	1	1	1	2	0	0	2	3	0	1	Neg	Neg	Neg	Neg	1	1	0	1	1	0
16	0	0	3	4	0	0	3	3	1	2	2	0	0	3	1	4	4	0	3	2	0	2	Neg	Neg	Neg	Neg	1	1	1	1	1	1
17	0	3	4	4	1	2	4	2	2	1	2	1	2	3	3	4	2	2	2	3	1	2	Neg	Neg	Equ	Neg	2	2	1	2	1	1
18	0	1	2	1	0	0	1	0	2	1	0	0	0	1	1	1	1	0	2	2	0	1	Neg	Neg	Neg	Neg	2	2	2	2	2	1

19	2	0	4	2	1	1	4	4	3	4	1	1	2	2	3	4	1	1	2	4	2	4	Pos	Neg	Pos	Neg	1	1	1	1	2	1
20	0	0	1	3	0	1	1	0	0	0	1	0	1	1	1	1	0	0	1	0	0	1	Neg	Neg	Neg	Neg	1	3	1	1	1	1

Abbreviations: AS, atrial septum; AVN, atrioventricular septum; E, endocardium; EL, endocardium in the left side; Equ, equivocal; ER, endocardium in the right side; I, interstitium; IHC, immunohistochemistry; LA, left atrium; LAA, left atrial appendage; Neg, negative; N/P, neural/perineural involvement; pCR, phenol Congo red; Pos, positive; RA, right atrium; RAA, right atrial appendage; SVC, superior vena cava; V, vessel

* Positive means positive deposition in the nerve; equivocal means positive deposition in the perineurium but negative deposition in the nerve.

Supplementary Table S2. Summary of the quantitative analysis results of ATTR deposition burden

Case #	VS	AS	RA	RAA	LA	LAA	SVC
1	0.515	0.067	0.209	0.03	0.832	0.291	0.682
2	10.488	15.214	0.821	0.459	10.866	18.486	10.561
3	1.011	0	0.114	0	0.601	0.017	0.023
4	5.33	0	0.572	0.038	0.304	1.175	0
5	15.768	12.275	3.917	3.383	9.758	6.139	0.776
6	9.643	4.879	0.657	0.148	8.986	5.588	7.159
7	9.16	2.374	0.723	0.243	1.728	3.425	1.473
8	13.323	14.949	0	0	14.506	12.534	1.179
9	0.126	0.174	0	0	0.574	0.104	0.104
10	23.985	17.264	15.247	3.998	3	13.228	12.045
11	2.029	0.808	0	0	13.752	0.579	0.005
12	24.87	23.509	0.354	1.09	18.431	7.355	4.396
13	0	20.828	0	0	7.077	16.469	18.695
14	0.025	0	0	0	0.143	0.095	0
15	2.264	6.059	0.388	0.66	1.669	1.758	0.46
16	8.711	7.135	6.685	0	18.308	14.014	6.015
17	17.164	16.947	0.62	6.149	13.969	4.658	5.774
18	5.346	1.024	0.155	0	1.439	3.595	0.128
18	24.166	19.217	19.897	7.109	16.785	7.123	16.983
20	0.031	0.18	0	0.134	0.054	0.074	0.077
Mean	8.7	8.1	1.2	2.5	5.8	7.1	4.3
SD	8.4	8.2	2.1	5.3	5.9	6.7	5.8
Min	0	0	0	0	0.05	0.02	0
Max	24.9	23.5	7.1	19.9	18.5	18.4	18.7

Abbreviation: SD, standard deviation

Supplementary Table S3. Summary of comparison of semiquantitative analysis results in right and left atria and atrial appendages.

Region		Right	Left	*P* value*
Semiquantitative ATTR deposition grading (range)				
Atrial septum	Endocardium	2.4 ± 1.6 (0–4)	0.6 ± 0.9 (0–3)	**<0.01**
Atrium	Endocardium	1.1 ± 1.3 (0–4)	1.7 ± 1.0 (0–3)	0.88
	Vessel	1.2 ± 1.1 (0–3)	1.5 ± 1.1 (0–4)	0.80
	Interstitium	1.1 ± 1.1 (0–4)	2.5 ± 1.3 (1–4)	**<0.01**
Atrial appendage	Endocardium	1.1 ± 1.1 (0–4)	1.2 ± 1.2 (0–4)	0.10
	Vessel	0.9 ± 1.3 (0–4)	1.0 ± 1.2 (0–4)	0.41
	Interstitium	0.8 ± 0.7 (0–2)	2.0 ± 0.9 (1–4)	**<0.01**
Semiquantitative AANF deposition grading (range)				
Region		Right	Left	*P* value
Atrium	Interstitium	1.4 ± 0.7 (0–3)	1.9 ± 0.9 (0–4)	0.28
Atrial appendage	Interstitium	1.9 ± 0.8 (0–3)	2.2 ± 0.8 (1–4)	0.10
Quantitative ATTR deposition burden (%)				
Region		Right	Left	*P* value
Atrium	Interstitium	2.5 ± 5.3 (0–19.9)	7.7 ± 6.8 (0.1–18.4)	**<0.01**
Atrial appendage	Interstitium	1.2 ± 2.1 (0–7.1)	5.8 ± 5.9 (0–18.5)	**<0.01**

Boldface signifies values that are statistically significant at $p < 0.05$.

* Right vs. Left, compared using the Mann-Whiteny U test.
